# Destabilization of chromosome 9 in transitional cell carcinoma of the urinary bladder

**DOI:** 10.1054/bjoc.2001.2154

**Published:** 2001-12

**Authors:** F Kimura, A R Florl, H-H Seifert, J Louhelainen, S Maas, M A Knowles, W A Schulz

**Affiliations:** Urologische Klinik, Center for Biological and Medical Research, Heinrich-Heine Universität, Moorenstrasse 5, Düsseldorf, D-40225, Germany; ICRF Cancer Medicine Research Unit, St James's University Hospital, Leeds, UK

**Keywords:** bladder cancer, chromosome instability, DNA methylation, LINE-1, tumour suppressor genes

## Abstract

The most frequent genetic alteration in transitional cell carcinoma of the urinary bladder (TCC) is loss of chromosome 9 which targets *CDKN2A* on 9p. The targets on 9q are not confirmed. Here, 81 advanced TCC specimens were investigated for loss of heterozygosity (LOH) and homozygous deletions (HD) on chromosome 9q using multiplex analysis of microsatellite markers. 41/81 tumours (51%) showed LOH on 9q, with LOH at all markers in 33 cases. Eight partial losses involved three regions in 9q12, 9q22.3, and 9q33– 9q34. No mutations were identified in the candidate tumour suppressor gene *DBCCR1* in three tumours showing restricted LOH at 9q32-33. 22% of the specimens had HD at *CDKN2A*, but no HD was found on 9q. Two tumours had lost 9p only and five 9q only. 9q LOH was not related to tumour grade or stage and present or absent with equal frequency in recurrent TCC. LOH on 9q correlated with the extent of genome-wide hypomethylation (*P* < 0.0001) which extended into satellite sequences located in 9q12 juxtacentromeric heterochromatin. While the high frequency of chromosome 9q loss in TCC may reflect destabilization of the chromosome related to hypomethylation of repetitive DNA, the data are compatible with the existence of tumour suppressor genes on this chromosome arm. http://www.bjcancer.com © 2001 Cancer Research Campaign


*British Journal of Cancer* (2001) **85**(12), 1887–1893
© 2001 Cancer Research Campaign
doi: 10.1054/ bjoc.2001.2154, available online at http://www.idealibrary.com on IDEAL

http://www.bjcancer.com

### Destabilization of chromosome 9 in transitional cell carcinoma of the urinary bladder

**F Kimura[1], AR Florl[1], H-H Seifert[1], J Louhelainen[3], S Maas[1], MA Knowles[3] and WA Schulz[1,2]**

[1]Urologische Klinik; and [2]Center for Biological and Medical Research, Heinrich-Heine Universität, Moorenstrasse 5, D-40225 Düsseldorf, Germany; and [3]ICRF Cancer Medicine Research Unit, St James's University Hospital, Leeds, UK

**Summary** The most frequent genetic alteration in transitional cell carcinoma of the urinary bladder (TCC) is loss of chromosome 9 which targets *CDKN2A* on 9p. The targets on 9q are not confirmed. Here, 81 advanced TCC specimens were investigated for loss of heterozygosity (LOH) and homozygous deletions (HD) on chromosome 9q using multiplex analysis of microsatellite markers. 41/81 tumours (51%) showed LOH on 9q, with LOH at all markers in 33 cases. Eight partial losses involved three regions in 9q12, 9q22.3, and 9q33– 9q34. No mutations were identified in the candidate tumour suppressor gene *DBCCR1* in three tumours showing restricted LOH at 9q32-33. 22% of the specimens had HD at *CDKN2A*, but no HD was found on 9q. Two tumours had lost 9p only and five 9q only. 9q LOH was not related to tumour grade or stage and present or absent with equal frequency in recurrent TCC. LOH on 9q correlated with the extent of genome-wide hypomethylation ($P < 0.0001$) which extended into satellite sequences located in 9q12 juxtacentromeric heterochromatin. While the high frequency of chromosome 9q loss in TCC may reflect destabilization of the chromosome related to hypomethylation of repetitive DNA, the data are compatible with the existence of tumour suppressor genes on this chromosome arm. © 2001 Cancer Research Campaign http://www.bjcancer.com

**Keywords**: bladder cancer; chromosome instability; DNA methylation; LINE-1; tumour suppressor genes

There is general agreement that loss of genetic material on chromosome 9 is the most frequent genetic alteration in transitional cell carcinoma of the urinary bladder (TCC). Chromosome arms 9p and 9q, often both, are lost entirely or in part (reviewed by Ørntoft and Wolf, 1998; Knowles, 1999). Consistent loss of a specific genomic region in tumour cells is usually considered as evidence for the presence of a tumour suppressor gene. Accordingly, available evidence indicates that loss on 9p targets the *CDKN2A* locus. First, in most TCC with loss of heterozygosity (LOH) on chromosome 9p, defects are detected in the remaining allele of *CDKN2A*, involving deletion, promoter hypermethylation, or, less frequently, point mutation (Kai et al, 1995; Orlow et al, 1995; Packenham et al, 1995; Williamson et al, 1995; Akao et al, 1997; Gonzalgo et al, 1998; Florl et al, 2000). Second, the gene encodes two distinct proteins involved in two separate, important pathways regulating cell proliferation and genomic stability (Quelle et al, 1995). Third, re-expression of one of the protein products, $p16^{INK4A}$, has been shown to cause cell cycle arrest in bladder carcinoma cells (Grim et al, 1997; Bender et al, 1998).

The issue regarding chromosome 9q is less clear-cut. The identification of tumour suppressor genes on 9q has been hampered by the fact that in many TCC LOH has been observed across the entire chromosome arm. Data accumulated from several laboratories on the rarer partial deletions suggests at least three rather large target regions (Habuchi et al, 1995; Hornigold et al 1999; Simoneau et al, 1996; Habuchi et al, 1997; Nishiyama et al, 1999; Ohgaki et al, 1999; van Tilborg et al, 1999; Simoneau et al, 2000; van Tilborg et al, 2000). Several genes located on chromosome 9q are good candidates from a functional point of view, e.g., *PTCH* or *TGFBRI*. For these, inactivation of the remaining allele by deletion, mutation, or hypermethylation at a significant frequency could not be found (Simoneau et al, 1996; McGarvey et al, 1998, 1999; Tokunaga et al, 1999). Instead, second events – with modest frequency – have been shown to inactivate the *TSC1* and *DBCCRI* genes whose functions in the urothelium are not understood (Habuchi et al, 1998; Hornigold et al, 1999; Nishiyama et al, 1999).

One alternative hypothesis accounting for the frequent loss of 9q in TCC is that it simply accompanies loss of 9p which is the event selected for. In support of this idea, loss of the entire chromosome is more frequent than that of 9q alone, and *CDKN2A* is firmly established as a potent tumour suppressor locus. However, as several reports describe more frequent loss of 9q in low grade superficial papillary TCC (Zhao et al, 1999; Chow et al, 2000), loss of 9q might be important for the development of papillary tumours. Loss of 9p, including homozygous deletion of *CDKN2*, is found at high frequency in muscle-invasive tumours as well. Since many of these do not develop from papillary precursors, it is possible that 9q LOH represents a passenger event accompanying 9p loss in these cases.

Another relevant consideration arises from the recognition that genomic alterations in tumours do not solely reflect selection for functional alterations, but also the underlying mechanisms of genetic instability. This notion has emerged, among others, from the study of HNPCC, in which certain types of mutation, e.g., microsatellite instability, are favoured as a consequence of the underlying defects in mismatch repair. Of many mutations occurring, only a few are functionally selected for. In contrast, some cases of colon cancer do not display microsatellite instability, but

*Received 30 April 2001*
*Revised 29 August 2001*
*Accepted 19 September 2001*

*Correspondence to:* WA Schulz chromosomal loss and rearrangements, indicating widespread chromosome instability (see Lindblom, 2001 for a recent review).

Conceivably, chromosome instability might not affect all chromosomes alike. For instance, DNA hypomethylation found in many human tumours favours instability of particular chromosomes. Hypomethylation of satellite 2 DNA sequences located in juxtacentromeric heterochromatin on chromosomes 1q and 16q correlates with loss of these chromosome arms in ovarian carcinoma and other tumours (Qu et al, 1999a,b; Saito et al, 2001). It is thought that hypomethylation of the satellite DNA favours recombination events thereby causing an increased propensity for chromosomal breaks (Tuck-Muller et al, 2000). Chromosome 9q12 contains another major region of juxtacentromeric heterochromatin, mainly consisting of satellite 3 DNA and other repeats such as MR9a. Since DNA methylation is diminished genome-wide in most cases of TCC (Jürgens et al, 1996; Florl et al, 1999), hypomethylation in the 9q12 region might account for the high frequency of chromosome 9q loss and explain why loss of the entire chromosome arm 9q predominates. This hypothesis predicts that loss of chromosome 9q in TCC is related to the extent of DNA hypomethylation.

To address these hypotheses we present a study of LOH on chromosomes 9p and 9q in – mostly advanced – TCC specimens, including mutational analysis of the candidate tumour suppressor gene *DBCCRI* and a correlation with DNA methylation levels.

### MATERIALS AND METHODS

### Patients and tissue samples

TCC samples were obtained by cystectomy, nephrectomy, or transurethral resection from 81 patients between 1994 and 1999. Tumours and corresponding normal tissues were dissected after surgery, frozen in liquid nitrogen, and stored at –80˚C. Blood samples from tumour patients were routinely collected for DNA extraction. Grading and staging were performed according to the 1997 UICC classification for bladder carcinoma. Among the 81 patients, 63 were male and 18 female. Ages ranged from 41 to 87 years with a mean age of 65.7 ± 9.1 years. In all, 5 tumours were staged as pTa, 8 carcinomas as pT1, 24 as pT2, 33 as pT3, and 11 as pT4. Three tumours were graded as G1, 23 as G2, 55 as G3, and 4 as G4, respectively. The study was favourably reviewed by the ethics committee of the Heinrich-Heine-University.

### DNA extraction

High molecular weight genomic DNA was isolated from frozen tissues and whole blood using the blood and cell culture DNA kit (Qiagen, Hilden, Germany).

### LOH analysis

Matched pairs of normal and tumour DNA samples were screened for LOH at 9q at the 14 microsatellite loci D9S1862, D9S273, D9S15, D9S153, D9S283, D9S196, D9S287, D9S180, D9S176, D9S53, D9S1872, D9S63, D9S1847, and D9S158 (cf Figure 1) using primer sequences obtained from the Genome DataBase. Testing of markers was performed in duplex or triplex PCR analyses repeated at least twice. Template DNA (100 ng) was amplified in a total volume of 50 µl containing 150 µM of each dNTP, 1.5 mM MgCl2, 10–100 pmol of each primer (forward primers modified at the 5′-end with IRD800), and 2 U of DyNAzymeII Taq polymerase (Biozym, Göttingen, Germany). Following initial denaturation at 95˚C for 5 min, 30–35 cycles of 30 s at 94˚C, 30 s at 55˚C or 60˚C, and 1 min at 72˚C were performed. All reactions included a final elongation step at 72˚C for 10 min. PCR products were analysed on an automated infrared DNA electrophoresis system (LI-COR dNA 4000/4200, MWG-Biotech, Ebersberg, Germany). Following addition of 4 µl of loading buffer to the 1:10-diluted PCR product, the sample was heated to 95˚C for 5 min and snap-cooled. From each mixture, 1.5 µl per lane was loaded onto a 6% denaturing polyacrylamide gel (SequaGel, National Diagnostics, Atlanta, Georgia) pre-run for 30 min at 1000 V. Electrophoresis was carried out at a constant voltage of 1000 V at 50˚C. The data are presented as an

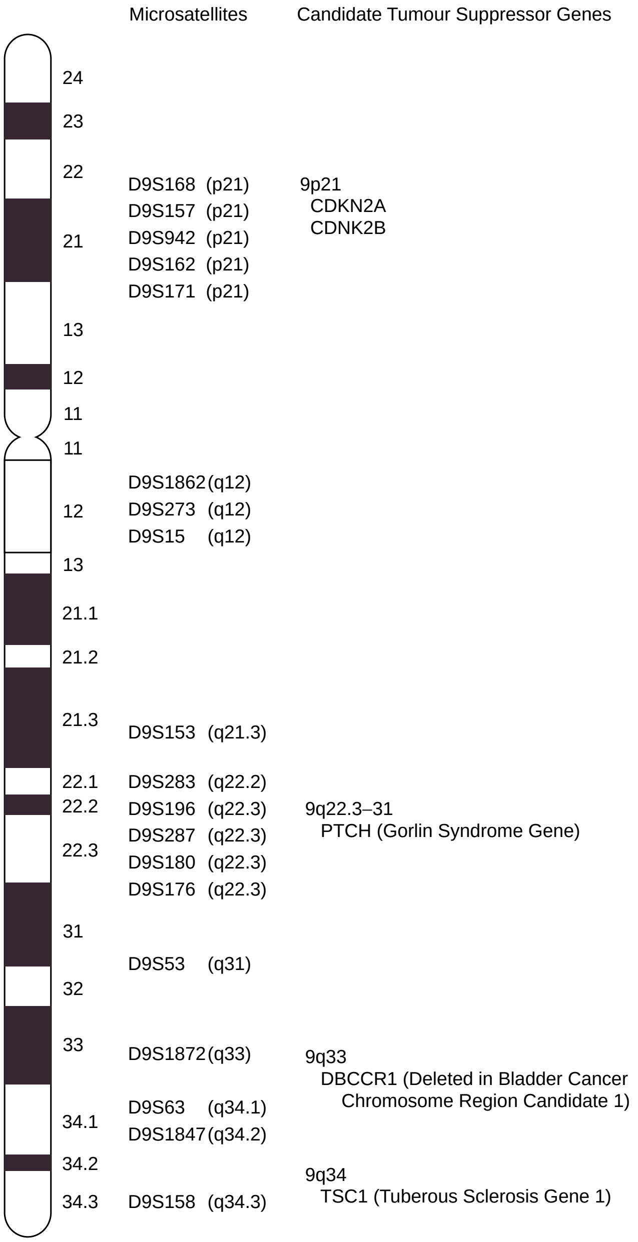

**Figure 1** Microsatellites and candidate tumour suppressor genes on chromosome 9. The order and approximate positions of microsatellites used in the study (left column) and the position of selected candidate tumour suppressor genes for TCC (right column) are indicated

*© 2001 Cancer Research Campaign*

radioautograph-like image that is stored in TIF format using the image manipulation subprogram of the LICOR Base ImagIR software package. Band analysis was performed using OneDScan 1.2 software (MWG-Biotech). An allele was regarded as lost when the intensity of the remaining signal was less than 60% of the signal from the same allele in the matching control DNA of the same patient, as previously described (Florl et al, 2000).

Homozygous deletions were identified by analyses of D9S942 microsatellite signals in multiplex reactions and confirmed by quantitative PCR of *CDKN2A* exons as described (Florl et al, 2000).

### Mutation analysis of *DBCCRI*

Ten pairs of primers designed to amplify the entire coding region of *DBCCRI* from genomic DNA were used (Habuchi et al, 1998). For exon 8 analysis, four pairs of primers were used to amplify the coding region as overlapping fragments. Primers were synthesized and labelled with FAM (sense primer) or TET (antisense) phosphoramidite dyes. PCR reactions were carried out under standard conditions. Cycle times were as follows: 95˚C for 4 min, 5 cycles of 95˚C for 60 s and 72˚C for 90 s followed by 25 cycles of 95˚C for 60 s, 55˚C for 60 s and 72˚C for 90 s. Products were combined with formamide and TAMRA-500 standards (PE Biosystems, Warrington, UK), denatured at 95˚C for 2 min and snap-cooled in iced water. Samples were loaded onto 36 cm well to read 6.5% (49:1) acrylamide, 5% glycerol gels on an ABI 377 DNA sequencer linked to an external circulating cooling water bath set at 18˚C. Run conditions were set at 50 W power limiting and gels run for up to 16 h. Data was collected using ABI Genescan 3.1 software and analysed using ABI Genotyper 2.5 software (PE Biosystems). Products from exons 8C and 8D were 426 and 436 bp, respectively. These products were also assessed by denaturing high performance liquid chromatography (DHPLC) using a Transgenomic WAVE HPLC (Transgenomic™) and DNASep column (Transgenomic™) as described (Kuklin et al, 1997). Buffer A contained 0.1 M triethylammonium acetate (TEAA). Buffer B contained 0.1 M TEAA and 25% acetonitrile. Analysis was carried out at a flow rate of 0.9 ml per min and a buffer B gradient increase of 2% per min for 4 min. Start and end concentrations of buffer B were determined empirically for each fragment. Eluation of DNA from the column was detected by absorbance at 260 nm. PCR fragments which showed abnormal migration of elution profiles were re-amplified using the corresponding non-labelled primer pair and sequenced (Big Dye Terminator kit, PE Biosystems).

### Methylation analysis

Genome-wide hypomethylation was analysed on Southern blots using a LINE-1 probe as described (Jürgens et al, 1996). In normal somatic tissues, LINE-1 sequences are almost completely methylated. Hypomethylation results in the appearance of lower size bands (0.5 to 4 kb) after digestion with *Hpa II*. The data are expressed as % hypomethylation, which is the intensity of these bands after *Hpa II* compared to *Msp I* digestion. The value in normal bladder tissue is 0.5–1%. Southern blot analysis was employed as well to determine satellite methylation using the pMR9a (Rocchi et al, 1991) probe kindly provided by Dr Rocchi, Bari, Italy.

### RESULTS

Eighty-one TCC specimens previously characterized for chromosome 9p loss (Florl et al, 2000) were further analysed for LOH using microsatellite markers spaced across chromosome 9q (Figure 1). Approximately one half of the tumours (41/81 = 51%) showed LOH on 9q (Table 1). LOH at all markers investigated was observed in 33 cases (41%), whereas eight showed partial losses (cases *a* –*h* in Figure 2). In this group, the partial losses occurred in three regions: the most centromeric region encompassed markers 9S1862 to 9S15, the second region centred around 9S176, and the most telomeric region involved 9S1872 to 9S158 (Figure 2, right).

In most cases (85%), LOH at all markers on 9q was accompanied by LOH of markers around *CDKN2A* on 9p; only five TCC with complete loss of 9q had retained all 9p markers and only two had 9p LOH only. All but one specimen with homozygous deletion in the *CDKN2* locus showed LOH for all markers on chromosome 9q; in one exceptional case (designated *h* in Figure 2) only the distal part of chromosome 9q was affected. This was one of three specimens with partial losses on 9q also displaying alterations on 9p.

Three samples (*c*, *g* and *h*) had discrete deletions that included the candidate tumour suppressor gene *DBCCRI* at 9q32–33 and were therefore screened for mutations on the retained allele. No tumour-specific sequence alterations were found.

Since apparent retention of homozygosity within a region of LOH is a useful indicator for homozygous deletions in tumour

**Table 1** LOH on chromosome 9q in relation to clinical and molecular parameters

| | 9q retention (%) | | 9q partial loss (%) | | 9q complete loss (%) | | Total (%) | |
|---|---|---|---|---|---|---|---|---|
| pTa + pT1 | 6 | (46.2) | 1 | (7.7) | 6 | (46.2) | 13 | (100) |
| pT2 – pT4 | 34 | (50.0) | 7 | (10.3) | 27 | (39.7) | 68 | (100) |
| G1 | 2 | (100) | 0 | (0) | 0 | (0) | 2 | (100) |
| G2 | 9 | (39.1) | 3 | (13.0) | 11 | (47.8) | 23 | (100) |
| G3 | 29 | (51.8) | 5 | (8.9) | 22 | (39.3) | 56 | (100) |
| N0 and M0 | 28 | (45.9) | 8 | (13.1) | 25 | (41.0) | 61 | (100) |
| N + or M + | 12 | (60.0) | 0 | (0) | 8 | (40.0) | 20 | (100) |
| 9p intact | 38 | (79.2) | 5 | (10.4) | 5 | (10.4) | 48 | (100) |
| 9p abnormal | 2 | (6.1) | 3 | (9.1) | 28 | (84.8) | 33 | (100) |
| *CDKN2A* HD | 0 | (0) | 1 | (5.6) | 17 | (94.4) | 18 | (100) |
| Recurrence with progression | 4 | (50.0) | 0 | (0) | 4 | (50.0) | 8 | (100) |
| Recurrence without progression | 4 | (50.0) | 0 | (0) | 4 | (50.0) | 8 | (100) |
| Total | 40 | (49.4) | 8 | (9.9) | 33 | (40.7) | 81 | (100) |

*© 2001 Cancer Research Campaign* 

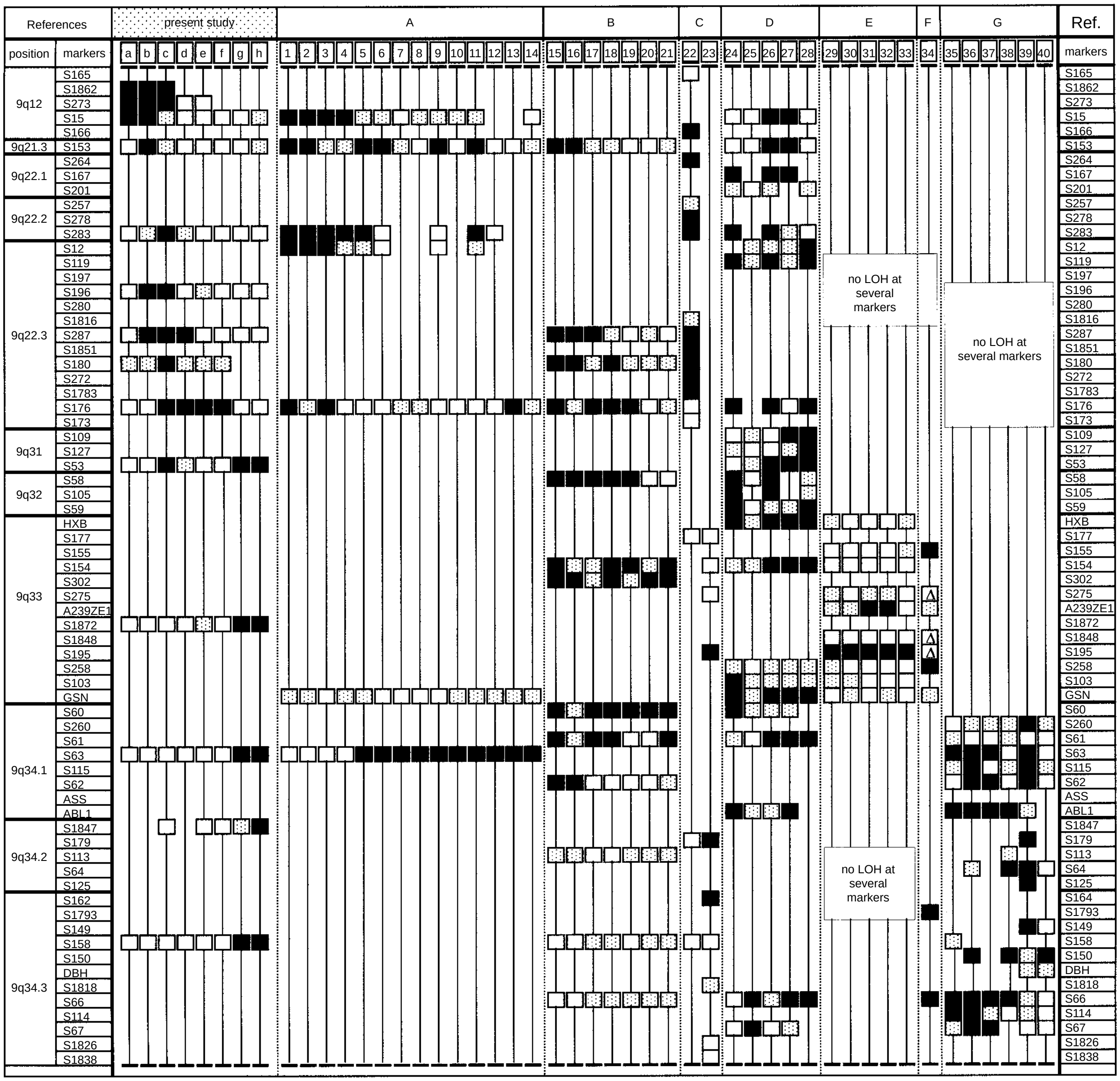

**Figure 2** Partial LOH on chromosome 9q in TCC. The figure shows a compilation of partial deletions on chromosome 9q mapped in the present study (cases a–h) and published in the literature (A: Simoneau et al, 1996, B: Ohgaki et al, 1999, C: van Tilborg et al, 1999, D: Habuchi et al, 1995, E: Habuchi et al, 1997, F: Nishiyama et al, 1999, G: Hornigold et al, 1995). Microsatellite markers, their order and approximate position are indicated. Squares black: LOH; white: retention, dotted: not informative, triangle: homozygous deletion. DBCCR1 is located near S195

tissue samples containing contaminating normal tissue (cf Florl et al, 2000), all such instances were carefully rechecked in repeated multiplex analyses. However, no homozygous deletion on chromosome 9q could be confirmed, even though at least 18 (22%) of the specimens in the series contain homozygous deletions in the *CDKN2A* locus on chromosome 9p (Table 1, cf Florl et al, 2000).

The presence of chromosome 9q alterations was neither related to tumour stage nor to tumour grade nor to the presence of metastases (Table 1). On the contrary, for every stage and grade the proportion of tumours with 9q LOH ranged between 40% and 56% of the total. Partial loss on 9q seemed to occur more often in more advanced tumours, but due to its low frequency, this relationship could not be statistically evaluated. All but one of the tumours with homozygous deletions at *CDKN2A* had LOH across the entire 9q arm (Table 1).

A detailed medical history was available for 73 patients. Sixteen tumour specimens in this study were from recurrences with initial papillary tumours (pTa or pT1). Half of these had progressed in stage or grade. LOH on 9q was found in four specimens each from the progressive group and from the non-progressive group with four specimens each retaining all tested markers on 9q. No case of partial loss was found among these 16 specimens.

Most of the tumour specimens exhibited often very pronounced DNA hypomethylation (Figure 3), as measured by loss of methylated sites in LINE-1 retrotransposons distributed throughout the genome. DNA hypomethylation was observed in specimens with 9q retention as well as 9q loss, but was on average much more

*© 2001 Cancer Research Campaign*

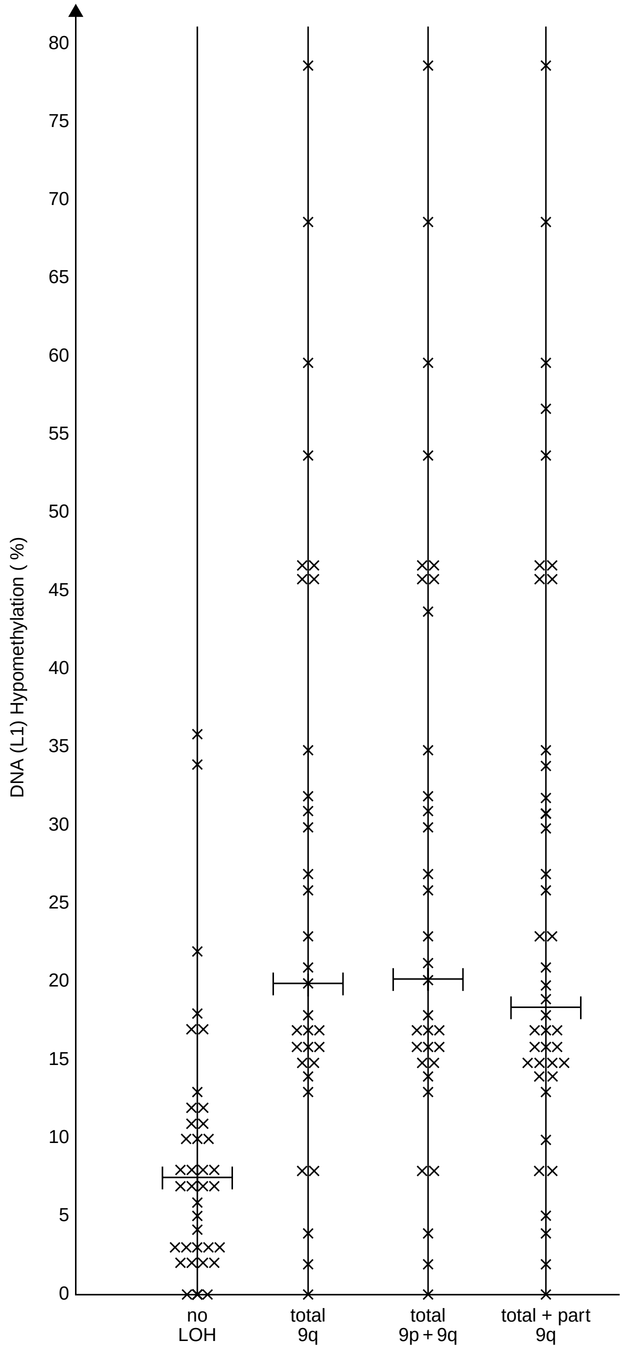

**Figure 3** 9q loss and DNA hypomethylation. The extent of genome-wide DNA hypomethylation as measured by decreased methylation of LINE-1 retrotransposon sequences is indicated for each TCC specimen. The nearly complete methylation of LINE-1 sequences in normal tissue is set at 0%. From left to right the columns show TCCs with (1) LOH on neither 9p nor 9q, (2) LOH at all studied markers on 9q, (3) LOH at all markers on 9q and/or markers on 9p (one case more than (2)), (4) LOH at any marker on 9q (eight more cases than (2)). Horizontal bars indicate median values pronounced in the latter group (mean ± S.D.: 27 ± 19% vs 10 ± 10%). This difference was highly significant ($P < 0.00001$ in a Wilcoxon sum analysis). In the eight tumours with partial 9q loss, DNA hypomethylation ranged from 5% to 57% with a mean ± S.D. of 20 ± 16% and was thus still more pronounced than in the specimens without alterations of chromosome 9.

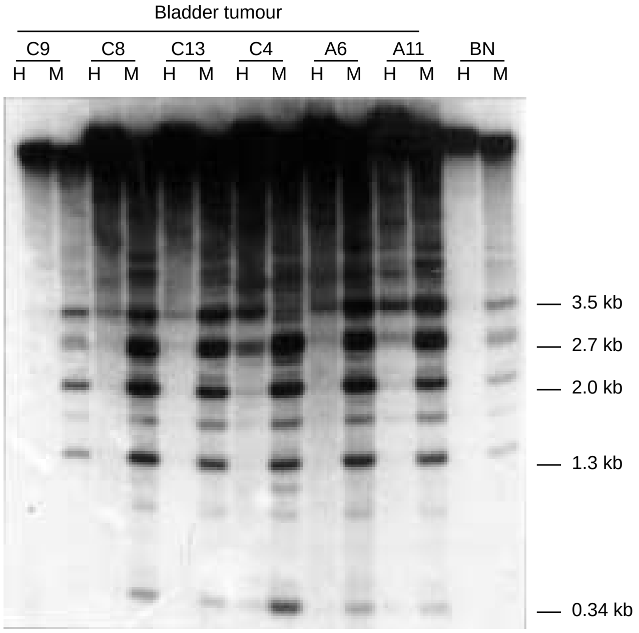

**Figure 4** Southern blot analysis of satellite sequence methylation. DNA from TCC specimens and from normal bladder was digested with *Hpa II* (H) or *Msp I* (M), blotted and hybridized with the pMR9a satellite probe. Approximate band sizes are indicated on the right hand side (note that they represent multiples of the 0.34 kb basic repeat). Because the enzyme recognition sequence is lacking in many repeats, most of the hybridization signal is present in the high molecular weight bands. Additional bands in the *Hpa II* lane compared to the *Msp I* lane indicate hypomethylation, most pronounced in specimen C4. The decreased intensity of the 3.5 kb band in the *Msp I* lane in the same specimen is due to a known polymorphism (Rocchi et al, 1991)

To obtain a more specific indication of the methylation status of repetitive sequences in the 9q juxtacentromeric region, 11 bladder cancer tissues previously characterised for LINE methylation were randomly selected and investigated by Southern hybridization with a chromosome 9 specific satellite probe (pMR9a) following DNA digestion with either of the restriction enzyme isoschizomers *Hpa II* and *Msp I* that are sensitive and non-sensitive to DNA methylation, respectively. As expected, the satellite sequence was strongly methylated in normal bladder tissue, as evidenced by a lack of lower size bands after *Hpa II* digestion as compared to *Msp I* digestion (Figure 4). In contrast, methylation of the satellite sequence was decreased in 7 of the 11 TCC tissues indicating that DNA hypomethylation also extends to the heterochromatic sequences (Figure 4).

### DISCUSSION

The results of the LOH analyses presented here agree with many other published studies in that a high percentage of TCC show losses across the entire long arm of chromosome 9 (in the present study 33/81 = 41%). Some of these studies have hinted at the possibility that loss of 9q might be an early step in TCC development and might be more frequent in papillary tumours. If so, losses of 9q in advanced tumours, which comprise the majority of cases in this study, ought to be often associated with a previous history of papillary tumours. Our data on patient history do not fit with this assumption, since recurrent TCC with a papillary tumour history displayed 9q LOH and retention at the same frequency. Likewise, the data contradict the assumption that LOH on 9q

© 2001 Cancer Research Campaign occurs essentially as a consequence of selection for *CDKN2A* inactivation. This hypothesis would predict that in advanced tumours 9p loss would be more frequent than 9q loss, whereas in our series localized LOH around *CDKN2A* was rather less frequent than LOH on 9q. Five cases showed LOH at all tested markers on 9q without alterations on 9p as compared to only two case of isolated 9p LOH. In addition, no changes around *CDKN2* were found in many cases with partial 9q losses.

While these findings are compatible with the idea that chromosome 9q carries one or several tumour suppressor genes contributing to TCC development, the identification of the relevant gene(s) has proved to be extremely difficult. In the present study, as in most others, only few tumours showed partial losses on 9q (8/81 = 10%). In the standard interpretation, the data (cf. Figure 2) would be taken to indicate tumour suppressor genes located in 9q12 (near 9S273), 9q22.3 (near S176), and more distally in 9q33–9q34. Although these locations do roughly correspond to those identified by other groups (see Figure 2), this interpretation should be applied with great caution. A detailed compilation of the published cases with partial 9q LOH (see Figure 2) rather suggests that partial losses can occur anywhere on chromosome 9q. This argument is strengthened by studies (Simoneau et al, 2000; van Tilborg et al, 2000) not included in the compilation that have reported a higher frequency of smaller deletions in up to five different regions of chromosome 9q, particularly in early stage tumours.

Since even moderately advanced stage TCC carry many different chromosomal alterations, it cannot be assumed that each one observed is functionally important. Rather, some low frequency changes may be functionally irrelevant but are carried along during expansion of the particular cell clone in which the events crucial for tumorigenesis and progression occurred. LOH and cytogenetic studies (Knowles et al, 1994; Kallioniemi et al 1995; Bruch et al, 1998; Richter et al, 1999; Fadl-Elmula et al, 2000) have shown that in TCC almost every chromosome is affected at a rate similar to that found for the frequency of partial chromosome 9q losses in this study (10%) and others. Moreover, in this study the partial losses tended to occur in advanced cancers. Taken together, there is no compelling reason to assume that individual cases of partial losses on chromosome 9q indicate the presence of a tumour suppressor gene target. Nevertheless, generalized chromosomal instability cannot explain why specifically loss of an entire chromosome 9q occurs so consistently in TCC.

As pointed out above, a consistent genetic alteration in a tumour can be a consequence of the particular mechanism driving genomic instability or of functional selection, or both. It seems likely that the preferential loss of chromosome 9q in TCC has a mechanistic cause. Chromosome 9, like chromosomes 1 and 16, contains an extended heterochromatic region juxtaposed to the centromere, composed mostly of satellite DNA. On chromosome 9, satellite 3 with sequences like pMR9a constitutes the bulk, whereas satellite 2 predominates on chromosomes 1 and 16. In the chromosome instability syndrome ICF, these satellites remain essentially unmethylated due to mutation of the DNA methyltransferase 3B. This causes unfolding of the juxtacentromeric heterochromatin and formation of unusual radial chromosomes from which chromosome arms involved, viz. 1p, 1q, 16p, or 16q, become lost (Xu et al, 1999). The same or a similar mechanism accounts probably for the loss of 1q or 16q in tumours with hypomethylated DNA (Tuck-Muller et al, 2000). Since our data demonstrate that 9q loss correlates with genome-wide hypomethylation (Figure 3), hypomethylation also affects 9q12 sequences (Figure 4) and LOH extends far into 9q12 (Figure 2 and data not shown), a related mechanism may also be involved in 9q loss in TCC. Radials formed from two chromosomes 9 could lose either 9q or 9p or both, the latter accounting for monosomy. Interestingly, one study (van Tilborg et al, 1998) has suggested that many cases of total chromosome 9 loss in TCC do not exhibit true monosomy, but rather losses of 9p from one chromosome and 9q from the other, which would be explained by the mechanism suggested here.

While instability of juxtacentromeric heterochromatin caused by DNA hypomethylation would explain the frequency of 9q loss and the predominance of loss of the entire arm, it does not account for the preferential loss of chromosome 9q as compared to 1q or 16q. Since, moreover, our data argue that 9p loss does not drive 9q loss, there is a strong argument for a tumour suppressor gene on 9q. However, the rarity of second hits by deletion on the remaining chromosome arm apparent from this and other studies suggests that this tumour suppressor may not conform to the standard two-hit-hypothesis. Since haploinsufficiency is one possible explanation, it will be important to determine the effect of loss of a single copy of candidate genes like *DBCCR1*, *PTCH*, or *TGFBRI*, singly or combined, on urothelial growth and differentiation.

### ACKNOWLEDGEMENTS

We are grateful to Ms Andrea Prior for technical assistance and to Ms C Steinhoff for help with the statistical analyses. We like to thank the Imperial Cancer Research Fund Mutation Detection Facility in Leeds for help with mutation analysis. The study was financially supported by the Deutsche Forschungsgemeinschaft and by the Forschungskommission der Heinrich-Heine-Universität Düsseldorf.

### REFERENCES

Akao T, Kakehi Y, Itoh N, Özdemir E, Shimizu T, Tachibana A, Sasaki MS and Yoshida O (1997) A high prevalence of functional inactivation by methylation modification of p16$^{INK4A}$/CDKN2/MTS1 gene in primary urothelial cancers. *Jpn J Cancer Res* **88**: 1078–1086

Bender CM, Pao MM and Jones PA (1998) Inhibition of DNA methylation by 5-aza-2′-deoxycytidine suppresses the growth of human tumor cell lines. *Cancer Res* **58**: 95–101

Bruch J, Wohr G, Hautmann R, Mattfeldt T, Bruderlein S, Moller P, Sauter S, Hameister H, Vogel W and Paiss T (1998) Chromosomal changes during progression of transitional cell carcinoma of the bladder and delineation of the amplified interval on chromosome arm 8q. *Genes Chromosom Cancer* **23**: 167–174

Chow NH, Cairns P, Eisenberger CF, Schoenberg MP, Taylor DC, Epstein JI and Sidransky D (2000) Papillary urothelial hyperplasia is a clonal precursor to papillary transitional cell bladder cancer. *Int J Cancer* **89**: 514–518

Fadl-Elmula I, Gorunova L, Mandahl N, Elfving P, Lundgren R, Mitelman F and Heim S (2000) Karyotypic characterization of urinary bladder transitional cell carcinomas. *Genes Chromosom Cancer* **29**: 256–265

Florl AR, Loewer R, Schmitz-Dräger BJ and Schulz WA (1999) DNA methylation and expression of L1 LINE and HERV-K provirus sequences in urothelial and renal cell carcinoma. *Br J Cancer* **80**: 1312–1321

Florl AR, Franke KH, Niederacher D, Gerharz CD, Seifert HH and Schulz WA (2000) DNA methylation and the mechanisms of CDKN2A inactivation in transitional cell carcinoma of the urinary bladder. *Lab Invest* **80**: 1513–1522

Gonzalgo ML, Hayshida T, Bender CM, Pao MM, Tsai YC, Gonzales FA, Nguyen HD, Nguyen TT and Jones PA (1998) The role of DNA methylation in expression of the p19/p16 locus in human bladder cancer cell lines. *Cancer Res* **58**: 1245–1252

Grim J, d'Amico A, Frizelle S, Zhou J, Kratzke RA and Curiel DT (1997) Adenovirus-mediated delivery of p16 to p16-deficient human bladder cancer cells confers chemoresistance to cisplatin and paclitaxel. *Clin Cancer Res* **3**: 2415–2423

 *© 2001 Cancer Research Campaign*

Habuchi T, Devlin J, Elder PA and Knowles MA (1995) Detailed deletion mapping of chromosome 9q in bladder cancer: evidence for two tumour suppressor loci. *Oncogene* **11**: 1671–1674

Habuchi T, Yoshida O and Knowles MA (1997) A novel candidate tumour suppressor locus at 9q32-33 in bladder cancer: localization of the candidate region within a single 840 kb YAC. *Hum Mol Genet* **6**: 913–919

Habuchi T, Luscombe M, Elder PA and Knowles MA (1998) Structure and methylation-based silencing of a gene (DBCCR1) within a candidate bladder cancer tumor suppressor region at 9q32-q33. *Genomics* **48**: 277–288

Hornigold N, Devlin J, Davies AM, Aveyard JS, Habuchi T and Knowles MA (1999) Mutation of the 9q34 gene TSC1 in sporadic bladder cancer. *Oncogene* **18**: 2657–2661

Jürgens B, Schmitz-Dräger BJ and Schulz WA (1996) Hypomethylation of L1 LINE sequences prevailing in human urothelial carcinoma. *Cancer Res* **56**: 5698–5703

Kai M, Arakawa H, Sugimoto Y, Murata Y, Ogawa M and Nakamura Y (1995) Infrequent somatic mutation of the MTS1 gene in primary bladder carcinomas. *Jpn J Cancer Res* **86**: 249–251

Kallioniemi A, Kallioniemi OP, Citro G, Sauter G, DeVries S, Kerschmann R, Caroll P and Waldman F (1995) Identification of gains and losses of DNA sequences in primary bladder cancer by comparative genomic hybridization. *Genes Chromosom Cancer* **12**: 213–219

Knowles MA, Elder PA, Williamson M, Cairns JP, Shaw ME and Law M (1994) Allelotype of human bladder cancer. *Cancer Res* **54**: 531–538

Knowles MA (1999) The genetics of transitional cell carcinoma: progress and potential clinical application. *BJU Int* **84**: 412–427

Kuklin A, Munson K, Gjerde D, Haefele R and Taylor P (1997) Detection of single-nucleotide polymorphisms with the WAVE DNA fragment analysis system. *Genet Test* **1**: 201–206

Lindblom A (2001) Different mechanisms in the tumorigenesis of proximal and distal colon cancers. *Curr Opin Oncol* **13**: 63–69

McGarvey TW, Maruta Y, Tomaszewski JE, Linnenbach AJ and Malkowicz SB (1998) PTCH gene mutations in invasive transitional cell carcinoma of the bladder. *Oncogene* **17**: 1167–1172

McGarvey TW, Tait E, Tomaszewski JE and Malkowicz SB (1999) Expression of transforming growth factor-beta receptors and related cell-cycle components in transitional-cell carcinoma of the bladder. *Mol Urol* **3**: 371–380

Nishiyama H, Takahashi T, Kakehi Y, Habuchi T and Knowles MA (1999) Homozygous deletion at the 9q32–33 candidate tumor suppressor locus in primary human bladder cancer. *Genes Chromosom Cancer* **26**: 171–175

Ohgaki K, Minobe K, Kurose K, Iida A, Habuchi T, Ogawa O, Kubota Y, Akimoto M and Emi M (1999) Two target regions of allelic loss on chromosome 9 in urinary-bladder cancer. *Jpn J Cancer Res* **90**: 957–964

Orlow I, LaRue H, Osman I, Lacombe L, Moore L, Rabbani F, Meyer F, Fradet Y and Cordon-Cardo C (1999) Deletions of the INK4A gene in superficial bladder tumors. *Am J Pathol* **155**: 105–113

Ørntoft TF and Wolf H (1998) Molecular alterations in bladder cancer. *Urol Res* **26**: 223–233

Packenham JP, Taylor JA, Anna CH, White CM and Devereux TR (1995) Homozygous deletions but no sequence mutations in coding regions of p15 or p16 in human primary bladder tumors. *Mol Carcinog* **14**: 147–151

Qu GZ, Grundy PE, Narayan A and Ehrlich M (1999a) Frequent hypomethylation in Wilms tumors of pericentromeric DNA in chromosomes 1 and 16. *Cancer Genet Cytogenet* **109**: 34–39

Qu G, Dubeau L, Narayan A, Yu MC and Ehrlich M (1999b) Satellite DNA hypomethylation vs. overall genomic hypomethylation in ovarian epithelial tumors of different malignant potential. *Mutat Res* **423**: 91–101

Quelle DE, Zindy F, Ashmun RA and Sherr CJ (1995) Alternative reading frames of the INK4a tumor suppressor gene encode two unrelated proteins capable of inducing cell cycle arrest. *Cell* **83**: 993–1000

Richter J, Wagner U, Schraml P, Maurer R, Alund G, Knonagel H, Moch H, Mihatsch MJ, Gasser TC and Sauter G (1999) Chromosomal imbalances are associated with a high risk of progression in early invasive (pT1) urinary bladder cancer *Cancer Res* **59**: 5687–5691

Rocchi M, Archidiacono N, Ward DC and Baldini A (1991) A human chromosome 9-specific alphoid DNA repeat spatially resolvable from satellite 3 DNA by fluorescent in situ hybridization. *Genomics* **9**: 517–523

Saito Y, Kanai Y, Sakamoto M, Saito H, Ishii H and Hirohashi S (2001) Expression of mRNA for DNA methyltransferases and methyl-CpG-binding proteins and DNA methylation status on CpG islands and pericentromeric satellite regions during human hepatocarcinogenesis. *Hepatology* **33**: 561–568

Simoneau AR, Spruck CH 3rd, Gonzalez-Zulueta M, Gonzalgo ML, Chan MF, Tsai YC, Dean M, Steven K, Horn T and Jones PA (1996) Evidence for two tumor suppressor loci associated with proximal chromosome 9p to q and distal chromosome 9q in bladder cancer and the initial screening for GAS1 and PTC mutations. *Cancer Res* **56**: 5039–5043

Simoneau M, LaRue H, Aboulkassim TO, Meyer F, Moore L and Fradet Y (2000) Chromosome 9 deletions and recurrence of superficial bladder cancer: identification of four regions of prognostic interest. *Oncogene* **19**: 6317–6323

Tokunaga H, Lee DH, Kim IY, Wheeler TM and Lerner SP (1999) Decreased expression of transforming growth factor beta receptor type 1 is associated with poor prognosis in bladder transitional cell carcinoma patients. *Clin Cancer Res* **5**: 2520–2525

Tuck-Muller CM, Narayan A, Tsien F, Smeets DF, Sawyer J, Fiala ES, Sohn OS and Ehrlich M (2000) DNA hypomethylation and unusual chromosome instability in cell lines from ICF syndrome patients. *Cytogenet Cell Genet* **89**: 121–128

Van Tilborg AA, Hekman AC, Vissers KJ, van der Kwast TH and Zwarthoff EC (1998) Loss of heterozygosity on chromosome 9 and loss of chromosome 9 copy number are separate events in the pathogenesis of transitional cell carcinoma of the bladder. *Int J Cancer* **75**: 9–14

Van Tilborg AAG, Groenfeld LE, van der Kwast TH and Zwarthoff EC (1999) Evidence for two candidate tumour suppressor loci on chromosome 9q in transitional cell carcinoma (TCC) of the bladder but no homozygous deletions in bladder tumour cell lines. *Br J Cancer* **80**: 489–494

Van Tilborg AAG, de Vries A, de Bont M, Groenfeld LE, van der Kwast TH and Zwarthoff EC (2000) Molecular evolution of multiple recurrent cancers of the bladder. *Hum Mol Genet* **9**: 2973–2980

Williamson MP, Elder PA, Shaw ME, Devlin J and Knowles MA (1995) p16 (CDKN2) is a major deletion target at 9p21 in bladder cancer. *Hum Mol Genet* **4**: 1569–1577

Xu GL, Bestor TH, Bourc'his, Hsieh CL, Tommerup N, Bugge M, Hulten M, Qu X, Russo JJ and Viegas-Pequinot E (1999) Chromosome instability and immunodeficiency syndrome caused by mutations in a DNA methyltransferase gene. *Nature* **402**: 187–191

Zhao J, Richter J, Wagner U, Roth B, Schraml P, Zellweger T, Ackermann D, Schmid U, Moch H, Mihatsch MJ, Gasser TC and Sauter G (1999) Chromosomal imbalances in noninvasive papillary bladder neoplasms (pTa). *Cancer Res* **59**: 4658–4661

*© 2001 Cancer Research Campaign*
